# Effects of Resistance Exercise in Older Adults With Sarcopenic Obesity: A Systematic Review and Meta-Analysis

**DOI:** 10.1097/jnr.0000000000000685

**Published:** 2025-07-01

**Authors:** Su-Ru Chen, Mei-Chuan Chen, Wen-Hsuan Hou, Pi-Chu Lin

**Affiliations:** 1 Post-Baccalaureate Program in Nursing and School of Nursing, College of Nursing, Taipei Medical University, Taipei, Taiwan; 2 Daan District Health Center, Taipei City; 3 Master Program in Long-Term Care, College of Nursing, Taipei Medical University, Taiwan; 4 Department of Physical Medicine and Rehabilitation, School of Medicine, College of Medicine, Taipei Medical University, Taipei, Taiwan; 5 Department of Physical Medicine and Rehabilitation, Taipei Medical University Hospital, Taipei, Taiwan; 6 School of Gerontology and Long-Term Care, College of Nursing, Taipei Medical University, Taipei, Taiwan; 7 Cochrane Taiwan, Taipei Medical University, Taipei, Taiwan; 8 Post-Baccalaureate Program in Nursing and Department of Nursing & Graduate Institute of Nursing, Asia University, Taichung, Taiwan; † Contributed equally

**Keywords:** body composition, meta-analysis, physical function, resistance exercise, sarcopenic obesity

## Abstract

**Background:**

In Taiwan’s aging society, age-related sarcopenic obesity has gradually become an important health issue. Sarcopenic obesity is associated with functional limitations, falls, disabilities, mental health problems, and even mortality.

**Purpose:**

The aim of this meta-analysis was to examine the effects of resistance exercise on body composition and physical functioning in older adults with sarcopenic obesity.

**Methods:**

A search of randomized controlled trials was conducted in six electronic medical databases from their inception to December 2023.

**Results:**

Twelve studies were included in the systematic review and meta-analysis, with results indicating a significant increase in grip strength (effect size=1.560, 95% confidence interval [CI]=[0.178, 2.941]; *p*=.027) and significant reduction in body fat percentage (effect size=−1.737, 95% CI=[−2.563, −0.912], *p*<.001) in the experimental group versus the control group. However, gait speed and body mass index were not significantly improved by resistance exercise interventions.

**Conclusions:**

The findings support a positive effect of resistance exercise on grip strength and body fat percentage in older adults with sarcopenic obesity.

## Introduction

Sarcopenic obesity (SO) is an increasingly important health problem and a cause of weakness in older adults (Kritchevsky, 2014). The prevalence of SO in older adults varies from 4% to 30% (Bahat et al., 2020; Gao et al., 2021; Stoever et al., 2018). Declines in muscle mass and physical activity reduce total energy expenditure and lead to excess adiposity that may lead to insulin resistance and metabolic syndrome (Alalwan, 2020). SO has been associated with a 24% increase in all-cause mortality risk (Tian & Xu, 2016; Zhang et al., 2019). It may also lead to higher risks of falls, disabilities, cardiovascular diseases, mental illness, institutionalization, and mortality (Batsis & Villareal, 2018; Chen et al., 2016; Fang et al., 2023). The most commonly proposed treatments currently focus on diet and exercise interventions (Colleluori & Villareal, 2021; Goisser et al., 2015). A combination of a hypocaloric diet with aerobic and resistance exercise has been proposed as the most effective method for improving physical function in obese older adults, although skeletal muscle mass is not completely preserved (Trouwborst et al., 2018; Xu et al., 2023). Aerobic exercise has little effect on fat mass in individuals with SO (Sawyer et al., 2015; Yin et al., 2020).

Resistance exercises are recommended as a non-pharmacological therapy for older adults with SO to minimize their sarcopenia and ameliorate the proinflammatory state associated with obesity. These exercises are defined as physical conditioning programs involving a variety of training modalities such as free weights, weight machines, and elastic bands that promote dynamic and static muscle contractions (Bouchonville & Villareal, 2013; Fleck & Kraemer, 2014). Resistance exercises can strike a balance among muscle strength, muscle power, and endurance to meet individual needs and enhance fitness, health, and sports performance (Carolyn & Lynn, 2012). A resistance training program has been shown to significantly increase muscle mass, reduce body fat, and increase muscle strength in older women (Liao et al., 2018) as well as improve gait speed and the functionality in older adults living in nursing homes (American College of Sports Medicine, 2010; Hortobágyi et al., 2015). Resistance training was recently reported to improve functional abilities and decrease sarcopenia severity in older adults (del Campo Cervantes et al., 2019). Also, Stoever et al. (2015) found that resistance training improves grip strength and gait speed in older adults with SO. The results of a meta-analysis conducted by Abbema et al. (2015) identified progressive resistance training at high intensities as the most effective exercise modality for improving preferred gait speed. In addition, several previous studies have reported the effect of resistance training on SO may be mediated by attenuating muscle mass loss, reducing the fat mass (Hsu et al., 2019; Huang et al., 2017), increasing grip strength (Hsu et al., 2019), and improving gait speed (Stoever et al., 2018).

Resistance exercise offers a potential treatment for age-related SO, with most related studies reporting resistance exercises to have positive effects on body composition and physical function in older adults with this condition (Eglseer et al., 2023). However, this treatment option is controversial, with some studies reporting resistance exercise as ineffective on SO (Chiu et al., 2018; Gadelha et al., 2016; Kim et al., 2016).

### Purpose

The results of treating older adults with SO have varied in related resistance exercise studies, with no consensus regarding the best types of exercise. Therefore, this study was developed as a systematic review with the goal of conducting a meta-analysis on the effects of resistance exercise on body composition and physical function in older adults with SO.

## Methods

A systematic review of randomized controlled trials (RCTs) with at least one control group was conducted using a meta-analytical approach. This systematic review was reported in accordance with the Preferred Reporting Items for Systematic Reviews and Meta-Analyses statement (Moher et al., 2009). The review protocol was registered a priori in the PROSPERO database of systematic reviews (www.crd.york.ac.uk/Prospero registration no. CRD42021274710).

### Search Strategy

EndNote X9 software was employed to manage the exported search data. Two authors independently conducted searches of the following electronic databases: CINAHL, PubMed, Cochrane Library, Embase databases, Airiti Library (Chinese Electronic Periodicals Service), and China National Knowledge Infrastructure from their inception to December 10, 2023. Any inconsistencies between the two authors were resolved via consensus with a third author. The following synonyms, MeSH terms, and Boolean Search keywords were used (aging OR old OR older OR elder OR elderly OR senior) AND (sarcopenia OR sarcopenic) AND (obese OR obesity) AND (resistance OR muscle OR strength OR weight) AND (exercise OR training).

### Inclusion and Exclusion Criteria

The inclusion criteria were (a) original articles with the full text available; (b) participants restricted to those ≥ 60 years of age with sarcopenia and obesity; (c) no limits on study language; and (d) randomized controlled trial or quasi-experimental study. The exclusion criteria were (a) combining two or more intervention measures so that a single effect could not be confirmed; (b) essays or retrospective literature; and (c) unable to obtain the complete full text.

### Quality Appraisal

The quality of the included studies was assessed using “the risk of bias in randomized trials” (RoB 2.0; Higgins et al., 2016). This assessment includes 21 signaling questions that address the domains of risk of bias arising from the randomization process, risk of bias due to deviations from the intended intervention or missing outcome data, risk of bias in measuring the outcome or in selecting the reported results, and overall bias. The permitted responses to each signaling question were: “yes,” “probably yes,” “probably no,” “no,” or “no information,” with each domain graded based on question responses as low, high, or of concern. Two reviewers assessed the methodological quality of the included articles independently using RoB 2.0 of the “Cochrane Handbook for Systematic Reviews of Interventions” (Higgins et al., 2019), and a third author was consulted to reach consensus on differences of opinion (Table 1).

**Table 1:** Risk of Methodological Bias Scores for the Included Studies (RoB 2.0)

Study	Randomization Process	Deviations from the Intended Intervention	Missing Outcome Data	Measurement of the Outcome	Selection of the Reported Results	Overall Bias
Banitalebi et al., 2020	U	U	L	L	L	U
Chen et al., 2017	L	L	L	L	L	L
Chiu et al., 2018	H	L	L	H	L	H
Cunha et al., 2018	L	L	L	L	L	L
Gadelha et al., 2016	L	L	L	L	L	L
Huang et al., 2017	L	L	L	L	L	L
Kim et al., 2016	L	U	L	H	L	H
Lee et al., 2021	L	L	L	L	L	L
Liao et al., 2017	L	L	L	L	L	L
Liao et al., 2018	L	L	L	L	L	L
Vasconcelos et al., 2016	L	U	L	L	L	U
Wang et al., 2019	U	U	L	L	L	U

*Note.* H = high risk of bias; L = low risk of bias; U = uncertain.

### Data Extraction

Data pertaining to the following variables were extracted: authors, country, study design, participants, setting, type of intervention, intervention duration, sample size, age, gender, and outcome variable. The primary outcome measure of interest was physical function (i.e., grip strength and gait speed), which is a key criterion for diagnosing sarcopenia. Body composition (i.e., percentage fat and body mass index [BMI]) was evaluated as a secondary outcome. The mean value and *SD* between baseline and final measures of the outcomes were extracted.

### Data Analysis

Treatment effects are presented in forest plots showing the effect sizes and 95% confidence intervals (CIs). Publication bias was examined using funnel plots, and the number of missing studies was calculated using Egger's test. The effect index was computed using Hedges’ g. (Higgins et al., 2016). Cochran’s Q and Higgins’ *I* ^2^ statistics were used to determine statistical heterogeneity among the included studies (Higgins et al., 2019). *I* ^2^ values of 25%, 50%, and 75% were respectively considered to indicate low, moderate, and high heterogeneity. A fixed-effects model was used when heterogeneity was low to moderate, and a random-effects model was used when heterogeneity was high (Borenstein et al., 2010). Furthermore, subgroup analyses were conducted based on study setting, intervention equipment used, and number of interventions performed. Meta-regressions were also performed to evaluate the linear relationship between effect size and treatment regimen. All statistics were computed using Comprehensive Meta-Analysis ver. 3.0 statistical software (Biostat, Englewood, NJ, USA).

## Results

### Literature Search

**Figure 1:**
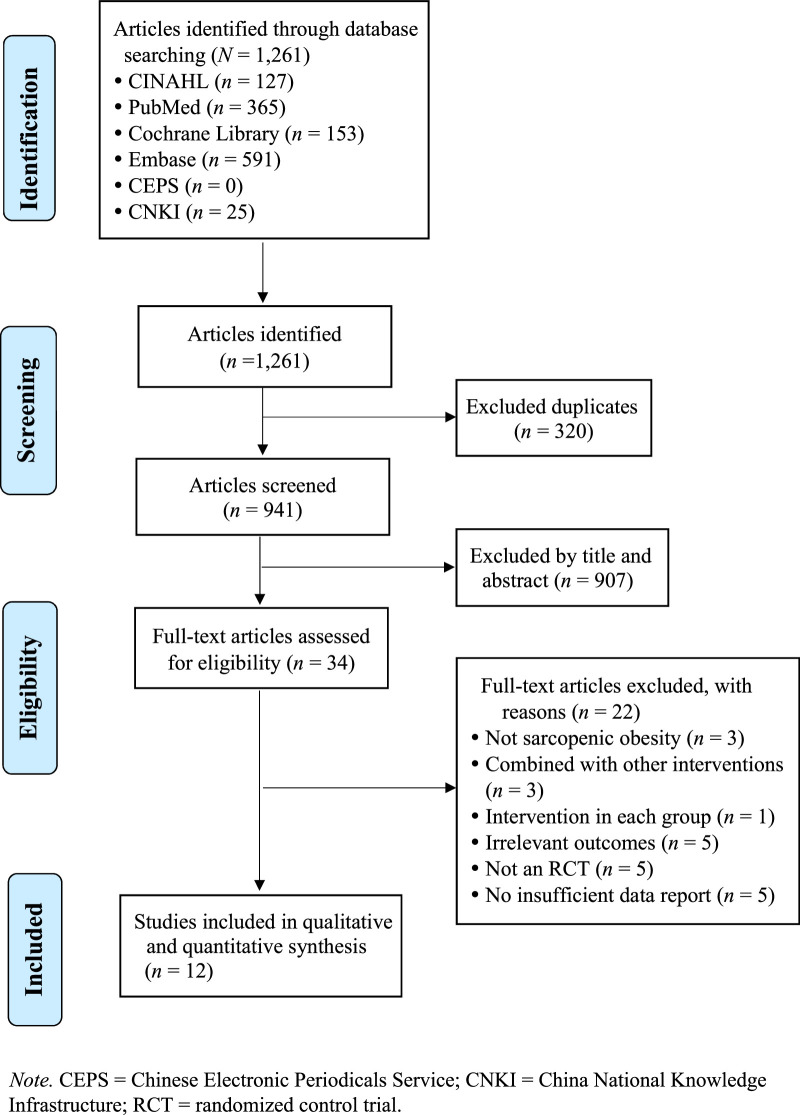
Meta-Analysis Article Selection Process

Of the 1,261 studies identified during the initial literature search, EndNote excluded 320 duplicates. After title and abstract screening, a further 941 were excluded, leaving 34 studies for full-text reading. Twenty-two studies were excluded after full-text reading due to resistance exercise interventions being applied to nonrelevant cases such as sarcopenia without obesity, dementia, cancer, diseases of the neuromusculoskeletal system, stroke, and cardiovascular diseases. The remaining 12 studies were subsequently included in the quality assessment and meta-analysis. Among these, 11 were randomized controlled studies (Chen et al., 2017; Huang et al., 2017; Lee et al., 2021; Liao et al., 2017; Liao et al., 2018; Cunha et al., 2018; Gadelha et al., 2016; Vasconcelos et al., 2016; Kim et al., 2016; Banitalebi et al., 2020; Wang et al., 2019), and one was a quasi-experimental study (Chiu et al., 2018; Figure 1).

### Study Characteristics

The characteristics of the 12 included studies are summarized in Table 2. Six were conducted in Taiwan (Chen et al., 2017; Chiu et al., 2018; Huang et al., 2017; Lee et al., 2021; Liao et al., 2017; Liao et al., 2018), three in Brazil (Cunha et al., 2018; Gadelha et al., 2016; Vasconcelos et al., 2016), and the remainder were conducted in Japan (Kim et al., 2016), Iran (Banitalebi et al., 2020), and China (Wang et al., 2019). Nine were conducted in community settings (Banitalebi et al., 2020; Chen et al., 2017; Cunha et al., 2018; Gadelha et al., 2016; Huang et al., 2017; Kim et al., 2016; Lee et al., 2021; Vasconcelos et al., 2016; Wang et al., 2019). The 12 studies covered a total of 632 participants aged 67–81 years. Nine of the studies included only women (Banitalebi et al., 2020; Cunha et al., 2018; Huang et al., 2017; Gadelha et al., 2016; Lee et al., 2021; Liao et al., 2017; Liao et al., 2018; Kim et al., 2016; Vasconcelos et al., 2016), with the remaining three including both men and women (Chen et al., 2017; Chiu et al., 2018; Wang et al., 2019). With regard to the intervention equipment used, elastic bands were used in six studies (Banitalebi et al., 2020; Huang et al., 2017; Lee et al., 2021; Liao et al., 2017; Liao et al., 2018; Vasconcelos et al., 2016), weight-training equipment was used in four (Chen et al., 2017; Cunha et al., 2018; Gadelha et al., 2016; Kim et al., 2016), sandbags were used in one (Chiu et al., 2018), and one study provided no related information (Wang et al., 2019). Intervention intensity in the 12 studies ranged from low to moderate, and the duration of the interventions ranged from 8 to 24 weeks.

**Table 2:** Summary of Included Studies

Study	Country	Study Design/Blinding	Participants/ Setting	Type of Intervention (Intensity)	Duration of Intervention	Control Group	Sample Size of Each Group (E/C)	Mean Age Mean±*SD* (y)	Women % (*n*/Total)	Outcome Variable
Banitalebi et al., 2020	Iran	RCT Single-blinded	Community	Elastic band	Three times a week for 12 wk	Usual care	32/31	E: 64.11±3.81 C: 64.05±3.25	100% (63/63)	Body composition
Chen et al., 2017	Taiwan	RCT Single-blinded	Community research center	Weight-training equipment (moderate)	Twice a week for 8 wk	Usual care	15/15	E: 68.9±4.4 C: 68.6±3.1	83% (25/30)	Body composition, grip strength
Chiu et al., 2018	Taiwan	Quasi-experimental design Non-blinded	Long-term care facilities	Sandbags (moderate)	Twice a week for 12 wk	Routine care	33/31	E: 79.64±7.36 C: 80.15±8.26	54.7% (35/64)	Body composition, physical function
Cunha et al., 2018	Brazil	RCT Single-blinded	Community	Weight-training equipment (moderate)	Three times a week for 12 wk	Usual care	20/21	E: 68.3±4.2 C: 67.3±3.6	100% (41/41)	Body composition, muscle strength
Gadelha et al., 2016	Brazil	RCT Single-blinded	Community	Weight-training equipment (moderate)	Three times a week for 24 wk	Usual care	69/64	E: 66.79±5.40 C: 67.27±5.04	100% (133/133)	Body composition
Huang et al., 2017	Taiwan	RCT Single-blinded	Community	Elastic band (low)	Three times a week for 12 wk	Routine care	18/17	E: 68.89±4.91 C: 69.53±5.09	100% (35/35)	Body composition
Kim et al., 2016	Japan	RCT Non-blinded	Community	Weight-training equipment (no information)	Twice a week for 12 wk	Routine care	35/34	E: 81.4±4.3 C: 81.1±5.1	100% (69/69)	Body composition, physical function
Lee et al., 2021	Taiwan	RCT Single-blinded	Community	Elastic band	Three times a week for 12 wk	Usual care	15/12	E: 70.13±4.41 C: 71.82±5.23	100% (27/27)	Body composition, physical function
Liao et al., 2017	Taiwan	RCT Double-blinded	Rehabilitation center	Elastic band (moderate)	Three times a week for 12 wk	Usual care	25/21	E:66.39±4.49 C:68.42±5.86	100% (46/46)	Body composition, physical function
Liao et al., 2018	Taiwan	RCT Single-blinded	Rehabilitation center	Elastic band (moderate)	Three times a week for 12 wk	Usual care	33/23	E: 66.67±4.54 C: 68.32±6.05	100% (6/56)	Muscle mass, physical function
Vasconcelos, et al., 2016	Brazil	RCT Single-blinded	Community	Elastic band (low)	Twice a week for 10 wk	Usual care	14/14	E: 72±4.6 C: 72±3.6	100% (28/28)	Physical function
Wang et al., 2019	China	RCT Single-blinded	Community	Progressive resistance training (moderate)	Twice a week for 8 wk	Usual care	20/20	E: 65.1±3.4 C: 64.1±2.8	47.5% (9/40)	Body composition, physical function

*Note.* E = experimental group; C = control group RCT = randomized control trial.

### Quality Assessment of the Studies

Nine studies met the standards for subject randomization (Chen et al., 2017; Cunha et al., 2018; Gadelha et al., 2016; Huang et al., 2017; Kim et al., 2016; Lee et al., 2021; Liao et al., 2017; Liao et al., 2018; Vasconcelos et al., 2016), two were affected by certain randomization concerns (Banitalebi et al., 2020; Wang et al., 2019), and one was deemed to have a high risk of bias (Chiu et al., 2018). Four of the studies were affected by a moderate risk of bias due to deviations from the initially stated intervention (Banitalebi et al., 2020; Kim et al., 2016; Vasconcelos et al., 2016; Wang et al., 2019). Ten of the studies had low risk of bias in their outcome measurements (Banitalebi et al., 2020; Chen et al., 2017; Cunha et al., 2018; Huang et al., 2017; Lee et al., 2021; Liao et al., 2017; Liao et al., 2018; Gadelha et al., 2016; Vasconcelos et al., 2016; Wang et al., 2019). All of the studies were assessed as having a low risk of bias in their reported results selection. The overall risk of bias assessment identified seven studies with a low risk (Chen et al., 2017; Cunha et al., 2018; Gadelha et al., 2016; Huang et al., 2017; Lee et al., 2021; Liao et al., 2017; Liao et al., 2018), two studies with a high risk (Chiu et al., 2018; Kim et al., 2016), and three studies with uncertain risk (Banitalebi et al., 2020; Vasconcelos et al., 2016; Wang et al., 2019).

### Effect on Grip Strength

**Figure 2:**
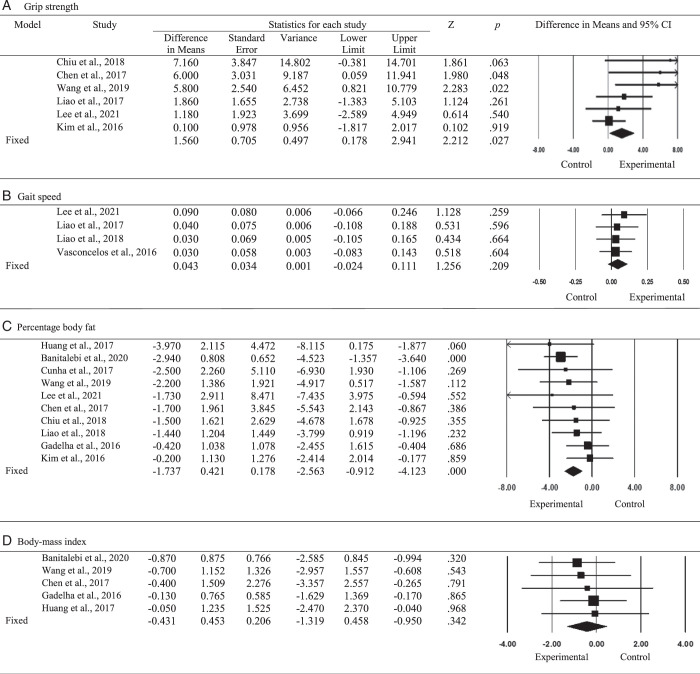
Effects of Resistance Exercise on Grip Strength, Gait Speed, Percentage Body Fat, and Body Mass Index

Calculating the *I* ^*2*^ and Cochrane *Q* statistics for grip strength identified lower heterogeneity in six of the trials (*Q*=9.352, *df*=5, *p*=.096, *I* ^*2*^=46.53). Thus, a fixed-effects model was employed, revealing an effect size (mean difference) of 1.560 (95% CI=[0.178, 2.941]; *p*=.027), indicating the presence of a significant effect of resistance exercise on grip strength (Figure 2A).

**Figure 3:**
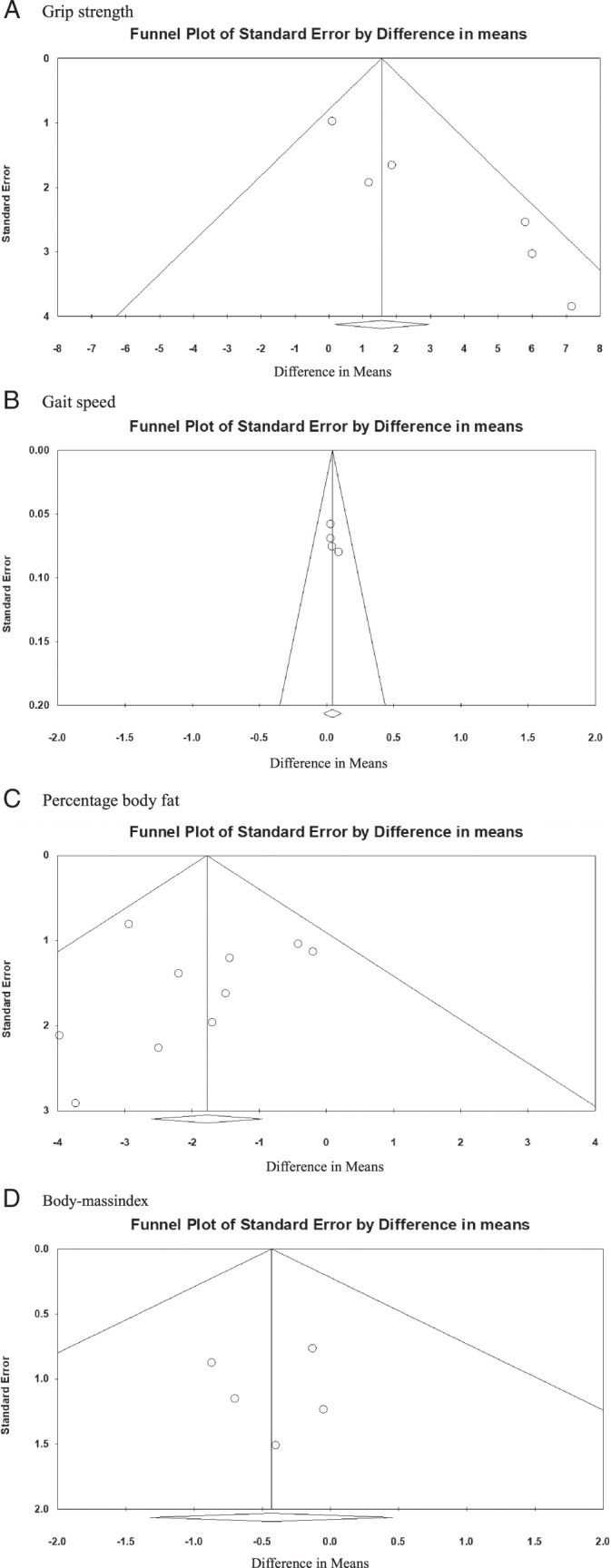
Publication Bias Related to the Grip Strength, Gait Speed, Percentage Body Fat, and Body Mass Index

Visual evaluation of the grip strength funnel plot revealed an asymmetrical phenomenon. The effect size in some studies was smaller than the average effect, and the intercept of the effect size of Egger’s regression was 2.724 (*t*=5.936; *p*=.004), while results of the Begg and Mazumdar test using Kendall’s tau with continuity correction were tau=0.400 and *Z*=1.127 (*p*=.259), indicating the presence of publication bias (Figure 3A).

### Effect on Gait Speed

Calculating the *I* ^*2*^ and Cochrane *Q* statistics for gait speed identified no heterogeneity among the four trials (*Q*=0.434, *df*=3, *p*=.933, *I* ^*2*^= 0). Thus, a fixed-effects model was employed, revealing an effect size (mean difference) of 0.043 (95% CI=[−0.024, 0.111]; *p*=.209), indicating no significant effect of resistance exercise on gait speed (Figure 2B).

As for publication bias, the funnel plot showed slight asymmetry and a lack of studies with larger sample sizes and negative results. The Egger test showed no publication bias (*p*=.13). The trim and fill method was not utilized because no publication bias was noted (Figure 3B).

### Effect on Percentage Body Fat

Calculating the *I* ^*2*^ and Cochrane *Q* statistics for percentage body fat identified minimal heterogeneity among the 10 trials (*Q*=7.101, *df*=9, *p*=.627, *I* ^*2*^= 0). Thus, a fixed-effects model was employed, revealing a significant reduction in percentage body fat and an effect size (mean difference) of −1.737 (95% CI=[−2.563, −0.912]; *p*<.001; Figure 2C).

As for publication bias, the funnel plot showed slight asymmetry and a lack of studies with larger sample sizes and negative results. The Egger test showed no publication bias (*p*=.43). The trim and fill method was not analyzed because no publication bias was noted (Figure 3C).

### Effect on Body Mass Index (BMI)

Results of the meta-analysis for BMI showed no heterogeneity among the five trials (*Q*=0.557, *df*=4, *p*=.968, *I* ^*2*^=0). Thus, a fixed-effects model was employed, revealing an effect size (mean difference) of −0.431 (95% CI=[−1.319, 0.458]; *p*=.342), indicating resistance exercise was not effective in reducing BMI (Figure 2D).

As for publication bias, the funnel plot showed slight asymmetry and a lack of studies with larger sample sizes and negative results. The Egger test showed no publication bias (*p*=.47). The trim and fill method was not analyzed because no publication bias was noted (Figure 3D).

### Subgroup and Meta-Regression Analyses

Subgroup analyses were conducted on the effect of grip strength using several variables, including study setting (i.e., community, other), gender (i.e., female only, both genders), equipment of the intervention (i.e., elastic band, weight training, and other), and number of interventions (i.e., ≤24 times and >24 times). Although the female-only subgroup revealed a mean lower effect of grip strength improvement after the intervention than the both gender groups (female only and both genders; *Q*=3.148, *p*=.076), all of the subgroups showed no significantly different effects of resistance exercise between the different subgroups (Table 3). A meta-regression analysis was also performed to further assess continuous variables such as mean age, percent female, number of interventions, publication year, and duration of intervention. However, no significant linear relationships were observed between effect sizes and treatment regimens in the included studies (Table 4).

**Table 3:** Subgroup Analyses of Resistance Exercise Effect on Grip Strength

Subgroup	No. Studies	Effect Size		Heterogeneity		
		Point Estimate (95% CI)	*p*	*Q*	*p*	*I* ^*2*^
Location of the study setting						
Community	4	1.251 [−0.309, 2.810]	.116	7.049	.070	54.77
Other	2	2.687 [−0.292, 5.667]	.077	1.601	.206	37.55
Total between			.106	0.744		
Type of gender						
Female only	3	0.161 [−0.164, 0.487]	.331	0.973	.331	0
Both genders	3	0.587 [0.247, 0.928]	.001	3.383	.001	0
Total between			.076	3.148		
Intervention equipment						
Elastic band	2	1.571 [−0.888, 4.029]	.210	0.072	.789	0
Weight training	2	0.656 [−1.168, 2.480]	.481	3.432	.064	70.86
Others	2	6.213 [2.058, 10.368]	.003	0.087	.768	0
Total between			.168	3.571		
No. interventions						
≤24	4	1.554 [−0.116, 3.225]	.062	9.280	.026	67.671
>24	2	1.571 [−0.888, 4.029]	.210	0.072	.789	0
Total between			.987	0.320		

*Note.* CI = confidence interval.

**Table 4:** Meta-Regression of the Effect of Resistance Exercise on Grip Strength

Item	Coefficient (95% CI)	*p*
Mean age	−0.023 [−0.056, 0.009]	.15
Female (%)	−0.008 [−0.018, 0.002]	.12
Number of intervention	−0.017 [−0.049, 0.015]	.29
Publication year	0.072 [−0.069, 0.215]	.31
Duration of intervention	−0.113 [−0.249, 0.022]	.10

## Discussion

This study was designed to explore the beneficial effects of resistance exercise on the grip strength and the percentage body fat in older adults with sarcopenic obesity. The primary outcomes of this meta-analysis included grip strength and gait speed, which are defining criteria used to clinically screen for sarcopenia (Chen et al., 2014). These criteria may be employed as predictors of health status and disability, institutionalization, and mortality risks in older adults. The other indicators, that is, BMI and percentage body fat, were identified as important indicators of obesity (Chen, 2018). Decreased physical activity in older adults is one of the causes of sarcopenic obesity (Allman et al., 2019). With population aging, the issue of sarcopenic obesity has received greater societal and research attention.

Resistance exercise refers to the use of resistance loading, different movement speeds, and various training forms to allow muscles to contract against resistance and practitioners to gradually increase muscle strength (Fleck & Kraemer, 2014). Resistance exercise has been shown to both reduce body fat and increase muscle strength (Liao et al., 2018).

The results of this study showed that resistance exercise can effectively increase grip strength in older adults with sarcopenic obesity. A meaningful, clinically important difference in grip strength has been defined as a decrease of 5.0–6.5 kg (Bohannon, 2019). The difference in means in three of the six studies included in this meta-analysis exceeded 5.0 kg. This result was similar to that of a study by Stoever et al. (2015) of an intervention consisting of progressive resistance training twice a week for 16 weeks, in which the right-hand-grip strength of older adults with sarcopenic obesity increased by 12%. Obesity exacerbates the physical capacity of individuals with sarcopenia. Therefore, resistance exercise is an appropriate exercise type to increase muscle strength. The subgroup analyses revealed no significant differences in the impact of resistance exercise on grip strength across these categories, Furthermore, the meta-regression analysis showed no significant linear relationships between different ages, genders, number of interventions, publication year, and duration of intervention. These findings suggest that the benefits of resistance exercise may make this intervention generally applicable for the improvement of muscle functioning in this population. However, the results also highlight a need for further research to explore potential moderators and mediators of intervention efficacy. Future studies should investigate other potential influencing factors that may moderate the effects of resistance training for sarcopenic obesity, such as nutritional status and baseline physical activity levels.

However, the findings of this study indicate that resistance exercise is not able to effectively improve gait speed in older adults with sarcopenic obesity. Bohannon (2014) showed the meaningful clinically important difference for change in comfortable gait speed of adults with pathologies to be 0.10–0.20 m s^−1^. The four studies included in our analysis that measured gait speed revealed a change of <0.1 m s^−1^. This result differs from Hortobágyi et al. (2015), who conducted a systematic review and meta-analysis that included 24 resistance exercise studies with 613 older adults and found gait speed increased 0.11 m s^−1^ or 9.3% with an effect size of 0.84. In addition, the findings of Van-Abbema et al. (2015) indicate high-intensity progressive resistance training to be the most effective training method for increasing gait speed. In this study, the majority of the included studies used low-intensity to moderate-intensity exercises and older adults as participants who may have performed the required exercises in a seated position. Therefore, increasing upper limb grip strength while failing to increase lower limb muscle strength resulted in no significant increase in walking speed.

The findings of this study also showed an effect of the resistance exercise interventions on reducing body fat. This result echoes that of a previous study that reported a significant association between exercise (aerobic exercises, resistance exercises, and exercise machines) and reduced percentage body fat (Moher et al., 2009). Kim et al. (2016) also found that, compared to a control group, resistance exercise reduces the body fat of older adults with sarcopenic obesity. Allman et al. (2019) found that resistance exercise contributing to an improved body composition was due in part to enhanced adipose tissue lipolysis and improved whole-body fat oxidation and energy expenditure in response to resistance exercise.

In this study, 91% of the participants were female. Compared to males, females are more likely to accumulate fat and have a higher percentage of body fat due to innate hormones and estrogen metabolism. Although the resistance exercise intensity of the included trials was mostly low to moderate, these levels were effective in reducing body fat. The American Heart Association recommends that training intensity in older adults should be maintained within a low to medium safety range and notes that older adults with sarcopenic obesity are often weaker and unable to perform high-intensity resistance exercises (Wu et al., 2014). Therefore, resistance training is recommended to reduce the percentage of body fat in older adults.

Notably, no effective reduction in BMI level was found in this study. The same finding was reported in a meta-analysis by Yin et al. (2020). This may be attributable to older adults reducing their percentage body fat but not losing weight through exercise.

As to adverse effects of resistance exercise reported in the 12 included studies, seven studies did not report on this issue, three reported no adverse effects (Lee et al., 2021; Liao et al., 2017; Liao et al., 2018), and two reported mild adverse effects (low back pain, musculoskeletal pain, and cramps in the lower limbs [Vasconcelos et al., 2016] and muscle soreness, knee pain, and shoulder pain [Banitalebi et al., 2020]).

There were some limitations in this study. First, only 12 trials were included, which is a small number. Because of sarcopenia, obesity has higher mortality and morbidity of cardiovascular diseases and metabolic diseases. More studies exploring the effects of exercise on physical performance are needed. Second, a few of the included trials used sample sizes of fewer than 30 participants in each group. Thus, inferences from these studies should be made with caution. Third, although subgroup and meta-regression analyses were performed, other potential confounding factors such as dietary habits and other exercises were not considered. Thus, the results of this meta-analysis should be interpreted with due caution. Finally, although the characteristics of the included studies were reviewed and it was concluded that most had satisfactory study design quality, three of the trials were identified as having moderate or high risks in their randomization processes, five as having a moderate risk of deviating from their initially stated interventions, and five as having an uncertain or high risk of overall bias. The difficulties faced in blinding subjects and researchers in this type of intervention process represent a major challenge in these types of studies.

### Conclusions

Sarcopenic obesity is gradually becoming a significant health problem. Exercise, especially resistance exercise, may improve grip strength and percentage body fat in older adults with this condition. However, further subgroup analyses and meta-regressions revealed no significant difference in the effects of resistance exercise between participants in different settings, using different intervention equipment, or participating in different numbers of sessions or treatment regimens.
